# The importance of experience: insights into optimal home-blood pressure monitoring regimens from the TASMINH4 Trial

**DOI:** 10.1097/HJH.0000000000004062

**Published:** 2025-05-23

**Authors:** Emily C. Morris, Katherine L. Tucker, Richard J. McManus, Richard J. Stevens

**Affiliations:** a Nuffield Department of Primary Care Health Sciences, University of Oxford, Oxford; b Brighton and Sussex Medical School, University of Brighton and University of Sussex, Brighton, UK

**Keywords:** blood pressure, hypertension, self-monitoring

## Abstract

**Objectives:**

This study investigates how prior home blood pressure monitoring (HBPM) experience affects blood pressure variability and evaluates if reduced HBPM regimens could be recommended for experienced patients.

**Methods:**

This posthoc analysis of the TASMINH4 trial included self-monitored blood pressure (BP) data from 225 patients. The standard deviation of systolic BP recordings was calculated for each patient-week to assess how BP variability changes with HBPM duration. A subgroup of 84 patients, who submitted at least 1 reading a day for 7 days at months 1, 3, and 6, was analysed to assess the impact of reduced HBPM regimens on BP estimates.

**Results:**

Day 1 readings were significantly higher than day 2–7 in the first 3 months of HBPM: 1.1 (95% CI 0.4, 1.8) day 1 vs. day 2. This effect diminished after 6 months: 1.0 (95% CI -0.8, 2.8) day 1 vs. 2. Long term monitoring significantly reduced intra-week BP variability, with the standard deviation of systolic BP recordings within each patient-week significantly reduced after 6 months. After 6 months of HBPM, the inclusion of day 1 readings or use of an abbreviated monitoring regimen had a reduced impact on estimates of mean systolic and diastolic blood pressure.

**Conclusions:**

Long-term HBPM reduces intra-week BP variability, making day 1 readings insignificantly raised after 6 months of HBPM. This provides rationale for different HBPM recommendations: longer regimes, excluding day one readings, for diagnosis and short-term monitoring; and abbreviated regimes including day 1 for longer term monitoring in those with HBPM experience.

## INTRODUCTION

Hypertension is a key risk factor for cardiovascular disease and stroke, the most important causes of morbidity and mortality worldwide [1]. In the UK, hypertension affects more than 13.5 million individuals and contributes to approximately 75 000 deaths annually [2]. Effective detection and management of hypertension requires accurate and consistent monitoring [2]. Home blood pressure monitoring (HBPM) has been shown to correlate more strongly with cardiovascular outcomes than office blood pressure measurements and is now recommended for both diagnosis and monitoring in national and international guidelines [3–6]. HBPM has become a routine part of hypertension management in primary care, with evidence supporting improved hypertensive control when self-monitoring is combined with professional and self-titration of antihypertensive therapy [7,8].

Despite evidence for HBPM efficacy, an international consensus on the optimal HBPM strategy has yet to be reached [1]. Current guidelines offer different recommendations regarding the number of days required for accurate assessment. The European Society of Cardiology (ESC) 2024 hypertension guidelines suggest that monitoring should continue beyond three days only if the average BP approaches the treatment threshold [6]. While Hodgkinson *et al.* (2019) made a similar recommendation [1], the ESC guidelines do not differentiate between diagnostic and long-term monitoring protocols, treating all self-monitoring periods similarly. In contrast, the NICE 2019 Hypertension Guidelines advocate for a 7-day monitoring period [2]. While Groenland *et al.* propose 4.5 consecutive days of HBPM, their study population had no prior monitoring experience and monitored for a total of 7 days [9].

A key consideration in HBPM protocols is the well documented “day 1 effect”, where systolic BP is systematically higher on the first day of monitoring, likely due to an initial alerting response [9–16]. With repeated short-term BP monitoring, recorded systolic blood pressure values are known to reduce in association with a fall in the alerting reaction [10,17]. It remains unclear whether this phenomenon persists with long-term self-monitoring. We hypothesise that with prolonged HBPM, BP variability decreases, leading to more stable and reliable measurements over time. If true, this could support shorter monitoring periods for long-term HBPM users and potentially challenge the necessity of excluding day 1 readings after patients have become accustomed to self-monitoring.

Utilising HBPM data collected from over 6 months, this study aimed to evaluate the impact of prior monitoring on blood pressure variability and assess whether a reduced HBPM regimen could be recommended for long-term monitors. Specifically, the study seeks to determine:

1. The mean blood pressure on each day of a monitoring week at month 1 and 6 of HBPM.
2. If day 1 readings are significantly higher than other days of the week after six months of HBPM.
3. How within patient-week blood pressure variability changes with duration of HBPM.
4. The mean BP obtained from reduced HBPM regimens compared to the NICE reference schedule [2].

## METHODS

### Study design and data source

This was a secondary analysis of the TASMINH4 trial, utilising self-monitored blood pressure data collected by the self- and telemonitoring intervention groups. The TASMINH4 trial was a multicentre randomised controlled trial investigating the efficacy of self-monitoring blood pressure, with or without telemonitoring, for hypertension management in primary care. The full trial methodology has been reported previously [8,18].

### TASMINH4 trial overview

The trial recruited patients with uncontrolled hypertension from 142 primary care practices in the United Kingdom. Eligibility criteria included age >35, baseline BP ≥140/90 and taking no more than three antihypertensive medications. Exclusion criteria included orthostatic hypotension, atrial fibrillation, dementia and chronic kidney disease grade ≥ 4. Eligible participants were randomly assigned (1 : 1 : 1) to three groups: GP antihypertensive titration using clinic readings (usual care), self-monitoring alone or self-monitoring with telemonitoring. Patients in the self- and telemonitoring groups were instructed to measure their BP in their nondominant arm, twice every morning and evening, for the first week of every month, for 12 months. BP readings were stored in device memory and downloaded for analysis at 6 and 12 months.

The TASMINH4 trial showed that self-monitoring, with or without telemonitoring, enabled general practitioners to adjust antihypertensive medication more effectively, resulting in significantly lower blood pressure compared to adjustments based on clinic readings alone [8,18].

### Study population

For this analysis, patients in the self- and telemonitoring groups who had submitted a minimum of 12 readings over at least two days at months 1, 3, and 6 were included. If patients provided more than one monitoring week per month, the first seven days were selected for consistency. A subgroup of 84 patients who submitted at least one BP reading per day for seven days at months 1, 3, and 6 was identified for assessing the impact of reduced monitoring regimens. Baseline trial data were matched to subsequent self-monitored blood pressure data using study IDs which were common in both datasets.

### Measurements

Daily systolic and diastolic BP readings were obtained from stored device memory.

### Statistical analysis

All statistical analyses were conducted using Stata 18 (Stata-Corp, College Station, Texas, USA).

The mean systolic BP recorded on day 1 was compared to subsequent days (days 2–7) in the first three months of home monitoring and after 6 months. Differences in mean systolic BP across days 1–7 were assessed using one-way analysis of variance (ANOVA), following verification of normality assumptions using skewness and kurtosis tests. Pairwise comparisons of mean BP between day 1 and subsequent days were performed with Bonferroni correction for multiple comparisons.

The standard deviation (SD) of systolic BP within each patient-week was calculated at months 1, 3, and 6 to assess variability over time. Changes in variability over time were assessed using one-way ANOVA.

BP estimates were compared across different monitoring regimens, including 7-day monitoring excluding day 1 (reference regimen, as per NICE guidance) [2], 7-day monitoring including day 1, 5-day monitoring (days 1–5), 5-day monitoring excluding day 1 (days 2–5), and 3-day monitoring (days 1–3). These analyses were conducted in the reduced dataset of 84 patients with sufficient data at months 1, 3, and 6. Mean systolic and diastolic BP values were calculated for each monitoring regimen. Differences between each regimen and the reference (7-day excluding day 1) were calculated using paired t-tests. Mean absolute differences were reported to assess the extent of variation from the reference regimen, with absolute values used to ensure all deviations, regardless of direction, were considered in the analysis.

### Ethical approval

Ethical approval for the original TASMINH4 trial was obtained from Oxford NHS Research Ethics Committee B (14/SC/0218). All patients gave written informed consent.

## RESULTS

**FIGURE 1:**
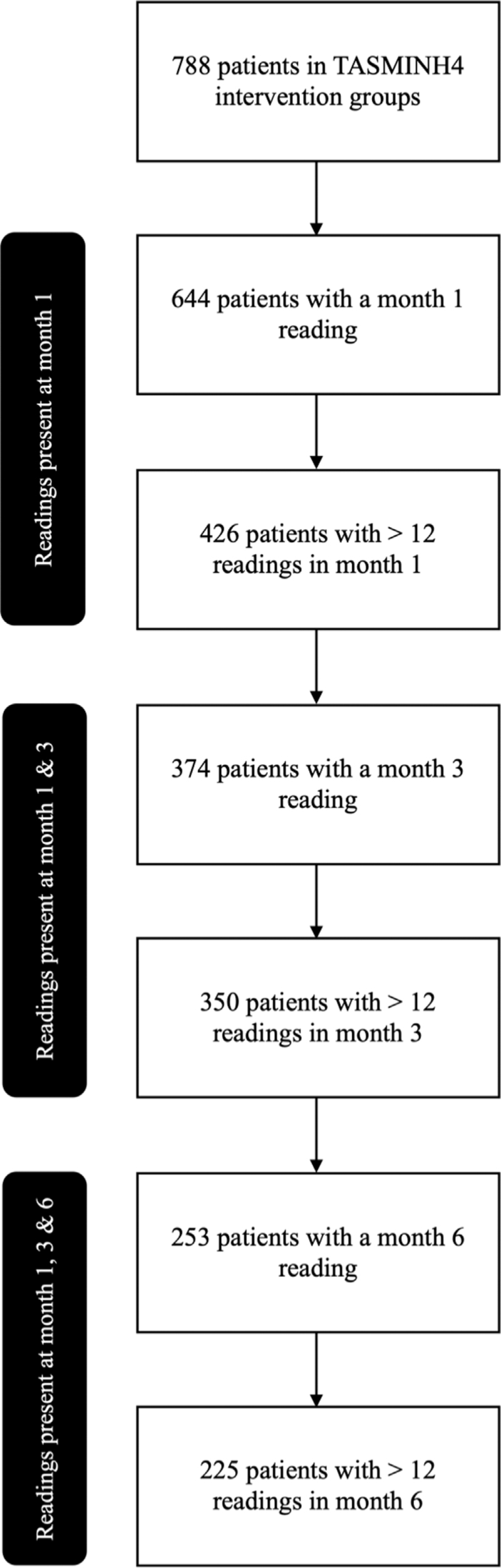
Flowchart identifying 225 patients with ≥12 readings at months 1, 3, and 6.

Of the 788 patients enrolled in the intervention groups of the TASMINH4 trial, 225 (29%) patients were included in this post-hoc analysis. Patients were required to have submitted a minimum of 12 readings, over at least two days at months 1, 3, and 6 for inclusion. Figure 1 highlights the sequential loss of patients at each stage of these inclusion criteria. The baseline characteristics of the resultant subgroup are shown in Table 1. Subgroup characteristics did not differ from those of the whole group participating in the TASMINH4 trial [8].

**TABLE 1:** Baseline characteristics of 225 patients and 84 patient subgroup included analysis, participants of the TASMINH4 clinical trial

	Self-monitoring group (*n* = 121, of 225 patient subgroup)	Telemonitoring group (*n* = 104, of 225 patient subgroup)	Self-monitoring group (*n* = 46, of 84 patient subgroup)	Telemonitoring group (*n* = 38, of 84 patient subgroup)
Age, mean (SD)	68.4 (8.1)	66.4 (9.5)	67.7 (7.6)	67.5 (8.8)
Systolic blood pressure (mmHg)	153.0 (13.0)	153.8 (13.2)	152.8 (13.8)	154.1 (12.6)
Diastolic Blood pressure (mmHg)	84.5 (9.5)	85.8 (9.4)	86.3 (9.8)	84.4 (8.2)
Sex				
Female	64 (52.9%)	50 (48.1%)	26 (56.5%)	19 (50.0%)
Male	57 (47.1%)	54 (51.9%)	20 (43.5%)	19 (50.0%)
Body-mass index (kg/m^2^)	29.1 (4.7)	28.9 (4.5)	29.0 (4.6)	28.1 (5.1)
Current smoker	3 (2.5%)	6 (5.8%)	2 (4.3%)	2 (5.3%)

Data are mean (SD) or *n* (%).

### Difference in BP readings across days of the week

Analysis using one-way ANOVA demonstrated that systolic blood pressure readings on day 1 were significantly higher than those recorded on days 2–7 during the first three months of HBPM. The mean systolic BP difference between day 1 and day 2 was 1.1 mmHg (95% CI: 1.8, 0.4), while the difference between day 1 and day 7 was 3.5 mmHg (95% CI: 4.5, 2.6) (Table 2, Supplemental Digital Content 1).

**TABLE 2:** Differences in mean systolic blood pressure between weekdays of HBPM

Month 1–3						
Difference in systolic blood pressure from weekday (mmHg)	Day 1	Day 2	Day 3	Day 4	Day 5	Day 6
Day 2	1.1 (0.4, 1.8) *(P* *<* *0.001)*					
Day 3	1.4 (0.7, 2.0) *(P* *<* *0.001)*	0.3 (-0.4, 1.0) *(P* *=* *1.000)*				
Day 4	1.7 (0.9, 2.3) *(P* *<* *0.001)*	0.6 (-0.2, 1.3) *(P* *=* *0.416)*	0.3 (-0.4, 1.1) *(P* *=* *1.000)*			
Day 5	2.6 (1.8, 3.3) *(P* *<* *0.001)*	1.5 (0.7, 2.2) *(P* *<* *0.001)*	1.2 (0.4, 2.0) *(P* *<* *0.001)*	0.9 (0.06, 1.7) *(P* *=* *0.034)*		
Day 6	2.2 (1.4, 3.0) *(P* *<* *0.001)*	1.1 (0.3, 1.9) *(P* *=* *0.001)*	0.9 (0.02, 1.7) *(P* *=* *0.054)*	0.5 (-0.3, 1.4) *(P* *=* *1.000)*	-0.3 (-1.2, 0.6) *(P* *=* *1.000)*	
Day 7	3.5 (2.6, 4.5) *(P* *<* *0.001)*	2.4 (1.5, 3.4) *(P* *<* *0.001)*	2.1 (1.2, 3.1) *(P* *<* *0.001)*	1.8 (0.8, 2.8) *(P* *<* *0.001)*	0.95 (-0.08, 2.0) *(P* *=* *0.137)*	1.3 (0.2, 2.4) *(P* *=* *0.08)*

Month 6						
Difference in systolic blood pressure from weekday (mmHg)	Day 1	Day 2	Day 3	Day 4	Day 5	Day 6
Day 2	1.0 (−0.8, 2.8) *(P* *=* *1.000)*					
Day 3	1.9 (0.06, 3.8) *(P* *=* *0.049)*	1.0 (-1.0, 2.9) *(P* *=* *1.000)*				
Day 4	2.3 (0.4, 4.2) *(P* *=* *0.010)*	1.3 (-0.7, 3.4) *(P* *=* *1.000)*	0.4 (-1.7, 2.5) *(P* *=* *1.000)*			
Day 5	3.5 (1.4, 5.5) *(P* *<* *0.001)*	2.5 (-0.3, 4.7) *(P* *=* *1.000)*	1.5 (-0.7, 3.8) *(P* *=* *1.000)*	1.2 (-1.2, 3.5) *(P* *=* *1.000)*		
Day 6	2.2 (-0.01, 4.4) *(P* *=* *0.071)*	1.2 (-1.0, 3.5) *(P* *=* *1.000)*	0.3 (-2.1, 2.6) *(P* *=* *1.000)*	-0.1 (-2.5, 2.3) *(P* *=* *1.000)*	-1.3 (-3.8, 1.3) *(P* *=* *1.000)*	
Day 7	2.4 (-0.2, 4.9) *(P* *=* *0.146)*	1.4 (-1.3, 4.0) *(P* *=* *1.000)*	0.4 (-2.3, 3.1) *(P* *=* *1.000)*	0.04 (-2.7, 2.8) *(P* *=* *1.000)*	-1.1 (-4.0, 1.7) *(P* *=* *1.000)*	0.1 (-2.8, 3.1) *(P* *=* *1.000)*

The table presents the difference in systolic blood pressure (BP) between monitoring days, calculated as the column day minus the row day. A positive value indicates that the systolic BP on the column day was higher than the BP on the row day, whereas a negative value means that BP on the column day was lower than on the row day. For example, a value of 1.1 mmHg in the day 1 vs. day 2 cell means that BP on day 1 was, on average, 1.1 mmHg higher than on day 2. Conversely, a value of -0.3 mmHg in the day 5 vs. day 6 cell means that BP on day 5 was 0.3 mmHg lower than on day 6. P-values from ANOVA calculations are displayed below differences. 95% confidence intervals are shown in brackets.

When the analysis was repeated after six months of HBPM, the difference between day 1 and days 2, 6, and 7 was no longer statistically significant. The mean differences were 1.0 mmHg (95% CI: -0.8, 2.8) for day 1 vs. day 2, 2.2 mmHg (95% CI: −0.01, 4.4) for day 1 vs. day 6 and 2.4 mmHg (95% CI: −0.2, 4.9) for day 1 vs. day 7 (Table 2, Supplemental Digital Content 1). While day 1 readings remained higher than those on days 3–5, there were no other significant differences in BP readings between any days of the week.

### Intraweek blood pressure reading variability

The standard deviation (SD) of SBP recordings within each patient-week at months 1, 3, and 6 is shown in Table 3. Compared to month 1, the SD was not significantly reduced at month 3 (−0.03 ± 0.05 (SE), *P* = 0.74). By contrast, after 6 months of HBPM, the mean SD was significantly reduced (−0.8 ± 0.05 (SE), *P* < 0.0001). There was a similar pattern when day 1 readings were excluded. Compared to month 1, the mean SD was significantly reduced after 6 months of HBPM (−0.08 ± 0.05 (SE), *P* < 0.0001), but not at month 3 (−0.06 ± 0.05 (SE), *P* = 0.14).

**TABLE 3:** Differences in mean systolic blood pressure and within-week standard deviation between month 1, 3 and 6

Month		Number of measurements	Mean SYS (mmHg)	Coefficient of variation	Mean of within-person SD mmHg	Difference in SD from month 1 (± SE)	*P*-value
1	All readings	5506	138.0 (137.5, 138.4)	12.7	11.8 (11.7, 11.9)	Not Applicable	
	Day 1 excluded	4643	137.8 (137.3, 138.3)	12.8	11.7 (11.6, 11.8)	Not applicable	
3	All readings	6699	136.5 (136.1, 136.9)	12.5	11.8 (11.7, 11.9)	−0.03 ± 0.05	0.74
	Day 1 excluded	5209	136.0 (135.5, 136.4)	12.4	11.7 (11.6, 11.8)	−0.06 ± 0.05	0.14
6	All readings	6388	134.3 (133.9, 134.7)	11.8	11.0 (10.9, 11.1)	−0.8 ± 0.05	<0.0001
	Day 1 excluded	4 926	133.9 (133.4, 134.3)	11.7	10.9 (10.8, 11.00)	−0.8 ± 0.05	<0.0001

Standard deviation reduces after 6 months of monitoring, for 225 patients with submitted readings at month 1, 3 and 6. Values shown in brackets reflect confidence intervals for estimates of mean SYS and mean SD.

### Assessment of different home blood pressure monitoring regimens

Table 4 shows the mean systolic and diastolic blood pressures at months 1 and 6 using different reading protocols in 84 patients with sufficient data. The baseline characteristics of this subgroup (Table 1) were broadly similar to the main trial population [8,18]. For each reduced monitoring regimen, the mean BP values are presented alongside the mean and absolute mean differences from the reference BP value. The reference BP is the mean of 7 days of readings with day 1 excluded, as per the 2019 NICE Guidance [2].

**TABLE 4:** Comparison of different HBPM monitoring protocols on mean systolic and diastolic blood pressure at month 1 and 6

		7 Days readings, with day 1 excluded	7 Days readings	5 Days readings	5 Days readings, with day 1 excluded	3 Days reading
Systolic Blood Pressure						
Month 1	Mean systolic blood pressure	135.8 (133.6, 138.1) (reference)	136.0 (133.8, 138.2)	136.1 (133.8, 138.4)	135.9 (133.5, 138.3)	136.9 (134.6, 139.3)
	Mean difference from reference	Not applicable	+0.16 (-0.26, 0.57)	+0.24 (-0.42, 0.91)	+0.06 (-0.44, 0.56)	+1.08 (0.03, 2.13)
	Mean absolute difference from reference	Not applicable	0.98 (0.62, 1.33)	2.19 (1.73, 2.67)	1.83 (1.52, 2.14)	3.66 (2.92, 4.39)
Month 6	Mean systolic blood pressure	132.7 (130.63, 134.72) (reference)	132.7 (130.73, 134.72)	132.7 (130.56, 134.75)	132.7 (130.56, 134.75)	133.0 (131.00, 134.97)
	Mean difference from reference	Not applicable	+0.07 (-0.15, 0.29)	+0.05 (-0.48, 0.45)	-0.01 (-0.45, 0.54)	+0.29 (-0.51, 1.08)
	Mean absolute difference from reference	Not applicable	0.82 (0.69, 0.95)	1.77 (1.46, 2.09)	1.68 (1.38, 1.98)	2.91 (2.41, 3.41)

Diastolic blood pressure						
Month 1	Mean diastolic blood pressure	81.1 (79.6, 82.7) (reference)	81.3 (79.8, 82.8)	81.4 (79.9, 83.0)	81.2 (79.6, 82.8)	81.2 (79.9, 83.1)
	Mean difference from reference	Not applicable	+0.17 (-0.05, 0.39)	+0.26 (-0.11, 0.62)	+0.44 (-0.23, 0.32)	+0.34 (0.19, 1.32)
	Absolute difference from reference	Not applicable	0.60 (0.42, 0.78)	1.26 (1.02, 1.52)	1.00 (0.84, 1.17)	1.97 (1.67, 2.27)
Month 6	Mean diastolic blood pressure	78.8 (77.37, 80.27) (reference)	78.9 (77.47, 80.34)	78.9 (77.25, 80.29)	78.8 (77.39, 80.34)	79.0 (77.51, 80.52)
	Mean difference from reference	Not applicable	+0.08 (-0.05, 0.22)	+0.04 (-0.33, 0.22)	-0.05 (-0.24, 0.33)	+0.19 (-0.24, 0.62)
	Absolute difference from reference	Not applicable	0.50 (0.42, 0.59)	1.09 (0.93, 1.25)	0.95 (0.76, 1.14)	1.62 (1.37, 1.87)

Data from 84 patients with sufficient data. The reference bp is the mean of 7 days of readings with day 1 excluded. Mean and absolute mean differences (95% confidence intervals) from the reference BP are shown for each protocol. A positive mean difference indicates a higher BP estimate than the reference, while absolute mean differences reflect variability in BP estimates.

At month 1, including day 1 readings had a minimal impact on estimated mean systolic and diastolic blood pressure for 7- and 5-day protocols, with mean differences of +0.16 mmHg (95% CI −0.26, 0.57) and +0.24 mmHg (95% CI −0.42, 0.91) respectively (Table 4). However, when using only 3 days of readings, the mean systolic BP was slightly but significantly higher than the reference: +1.08 mmHg (95% CI 0.03, 2.13). The absolute mean differences, ranging from 1.8 to 3.7 mmHg, indicate variability around these mean differences. Diastolic BP differences for different monitoring regimes followed a similar pattern (Table 4).

At month 6, the mean self-monitored systolic and diastolic blood pressures were lower across all monitoring protocols compared to month 1, reflecting overall BP reductions observed in the TASMINH4 trial [8]. At month 1, the mean BP of the reference 7-day regimen excluding day 1 was 135.8/81.1 mmHg, compared to 132.7/78.8 mmHg at month 6.

Within month 6 there were no clinically or statistically significant differences in systolic and diastolic BP between the reference 7-day regimen excluding day 1 and the 5- or 3-day protocols. The mean systolic BP differences were +0.05mmHg (95% CI −0.48, 0.45) for 5 days and +0.29 mmHg (95% CI −0.51, 1.08) for 3 days. For diastolic BP, the mean differences to reference were +0.04 mmHg (95% CI −0.33, 0.22) for the 5-day protocol and +0.19mmHg (95% CI −0.24, 0.62) for the 3-day protocol.

By month 6, the inclusion or exclusion of day 1 readings had no meaningful impact on BP estimates, for both systolic and diastolic BP (Table 4). The absolute mean differences were smaller than at month 1, ranging from 1.9–2.7 mmHg.

## DISCUSSION

This study demonstrates that for patients self-monitoring their blood pressure over time, intra-week reading variability slightly reduces with ongoing monitoring. After six months of home blood pressure monitoring (HBPM), there was almost no difference between a 7-day regimen excluding day 1 and simply averaging the first three days of readings. These findings challenge the necessity of extended monitoring periods beyond three days for long-term HBPM, except in cases where readings are close to treatment thresholds. In such cases, the European Society of Cardiology (ESC) 2024 guidelines already recommend extending measurements beyond three days [6]. Our results provide new evidence supporting a further distinction between short-term diagnostic monitoring and long-term monitoring strategies. Other qualitative work suggests that this would be welcomed by patients [1]. Given that hypertension, once diagnosed, is generally a lifelong condition, this would represent a significant reduction in burden over potential decades of monitoring [19].

Consistent with previous studies, we observed a systematic elevation of average SBP on day 1 compared to subsequent days [9–16]. This elevation has been cited as evidence for excluding day 1 readings when calculating average home BP [13,16], though this practice has been debated [20–22]. Some studies, such as Groenland *et al.*, argue that including day 1 is preferable as a reduction in the number of measurements increased the variability of average BP estimates [9]. Our findings confirm that this day 1 effect is most relevant in shorter monitoring regimens, where including day 1 leads to a small (1–2 mmHg) increase in estimated mean systolic and diastolic blood pressure. While this difference is minor, it may be clinically significant for patients near treatment or diagnostic thresholds.

We hypothesise that the physiological habituation of the alerting response explains the reduction in blood pressure variation with extended periods of HBPM. Similar declines in BP have been observed with repeated measurements within a single clinic visit [23]. This habituation effect suggests that long-term HBPM protocols could be safely shortened, incorporating day 1 readings without significantly altering treatment decisions. Implementing such abbreviated protocols could improve adherence and patient satisfaction, reducing the burden of prolonged BP monitoring over months or years [24].

A strength of this study is its use of a large dataset of routinely collected BP readings, allowing us to examine how blood pressure variability changes over time with prolonged self-monitoring. By including only patients with a minimum of 12 readings over at least two days at months 1, 3, and 6, we ensured that our findings reflect changes due to monitoring duration rather than differences in patient population. To our knowledge, this is the first study to longitudinally assess HBPM readings and variability over multiple months, rather than focusing on single-week BP variability alone.

However, this study has limitations. Changes in blood pressure variability over time may have been influenced by intensified antihypertensive treatment over time. Since our patient cohort was drawn from the intervention arms of the TASMINH4 trial, they may have received additional antihypertensive therapy each month, which we could not control for due to the lack of monthly treatment data. Additionally, our analysis of monitoring regimen lengths was based on a subset of 84 patients and these findings should therefore be validated in larger cohorts. Finally, although patients were recruited from multiple general practices, this study was still limited to a single trial population, highlighting the need for replication in other datasets [8].

Despite these limitations, this study provides new evidence that HBPM protocols should be tailored based on the duration of monitoring. While longer monitoring periods that exclude day 1 may be more appropriate for initial diagnosis and short-term assessments, our variability findings suggest that shorter regimens incorporating day 1 may be sufficient for long-term BP tracking. This distinction has important implications for simplifying HBPM schedules, enhancing patient adherence and reducing the burden of prolonged monitoring. Future research should evaluate the clinical impact of these reduced monitoring strategies to further refine HBPM guidelines.

## ACKNOWLEDGEMENTS

None.

Previous presentations: None.

Funding received for this work: There was no specific funding for this work. KT, RS and RM receive funding from the National Institute of Health and Care Research (NIHR) Applied Research Collaboration (ARC) Oxford and Thames Valley at the Oxford Health NHS Foundation Trust. The views expressed are those of the authors and not necessarily those of the NIHR, the NHS or the Department of Health.

Authors’ contributions: R.M. conceived the study with R.S. and E.M. E.M., R.S., K.T., and R.M. developed the protocols. EM undertook the analysis under supervision from R.S., R.M., and K.T. E.M. wrote the first draft of the article, which was subsequently edited and approved by all co-authors. All authors have read, provided critical revision, and approved the final version of the manuscript.

### Conflicts of interest

R.M. has received BP monitors for research use from Omron and is working with them to develop a telemonitoring system for use in primary care. He receives no personal payment for such work. The remaining authors have no disclosures.

## Supplementary Material

Supplemental Digital Content
