# Supplementary material for: The importance of experience: insights into optimal home-blood pressure monitoring regimens from the TASMINH4 Trial
Source: J Hypertens. 2025 May 23;43(8):1400–6. doi: 10.1097/HJH.0000000000004062 (PMC12237135; doi:10.1097/HJH.0000000000004062)

Supplemental Digital Content

Supplemental Digital Content 1 | Graph depicting differences between day 1 mean systolic blood pressure, and subsequent days of the week, in month 1-3 or month 6 of HBPM. Error bars show 95% confidence intervals for mean differences. Day 1 readings were significantly higher than day 2-7 in the first 3 months of HBPM. After 6 months of HBPM, day 1 was not significantly higher than day 2, 6 or 7.

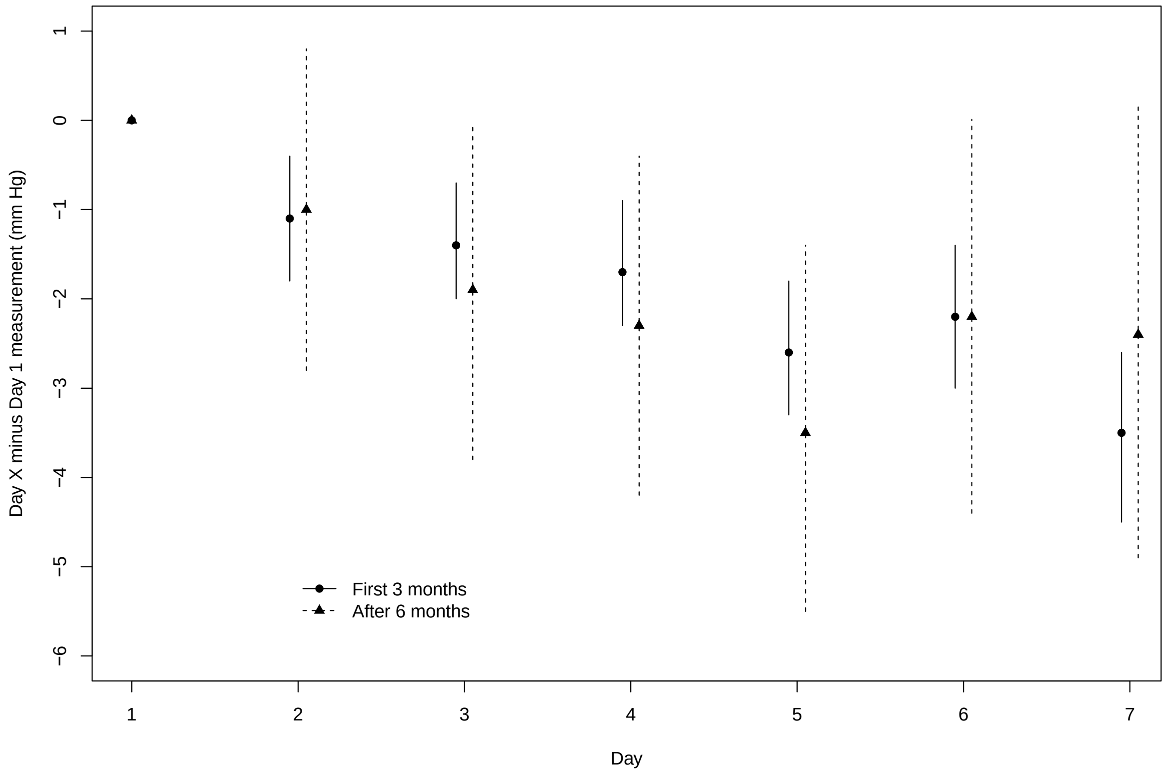

Supplement: Supplemental Digital Content [file jhype-43-1400-s001.pdf]
